# Comparison of Hemiarthroplasty, total hip arthroplasty, and internal fixation for hip fractures in patients over eighty years of age: factors affecting mortality: a nationwide cohort study of fifty three thousand, four hundred and ninety five patients from Türkiye

**DOI:** 10.1007/s00264-025-06412-8

**Published:** 2025-02-04

**Authors:** Kadir Uzel, Murat Birinci, Ömer Serdar Hakyemez, Bilal Bostanci, İzzet Bingöl, Umut Öktem, Naim Ata, M. Mahir Ülgü, Şuayip Birinci, Vasfi Karatosun, Bülent Atilla, İbrahim Azboy

**Affiliations:** 1 Faculty of Medicine, Department of Orthopaedics and Traumatology, İstanbul Medipol University, İstanbul, Türkiye; 2 Department of Orthopaedics and Traumatology, Tekirdağ Çerkezköy State Hospital, Tekirdağ, Türkiye; 3 Department of Orthopaedics and Traumatology, Şırnak State Hospital, İstanbul, Türkiye; 4 Faculty of Medicine, Department of Orthopaedics and Traumatology, Ankara Yildirim Beyazit University, Ankara, Türkiye; 5 Department of Orthopaedics and Traumatology, Ankara City Hospital, Ankara, Türkiye; 6 Ministry of Health, General Directorate of Health Information Systems, Ankara, Türkiye; 7 Ministry of Health, Deputy Minister, Ankara, Türkiye; 8 Department of Orthopaedics and Traumatology, İzmir Dokuz Eylül University, Faculty of Medicine, İzmir, Türkiye; 9 Department of Orthopaedics and Traumatology, Hacettepe University, Faculty of Medicine, Ankara, Türkiye

**Keywords:** Hip fracture, Mortality, Elderly, Hemiarthroplasty, Total hip arthroplasty, Internal fixation

## Abstract

**Purpose:**

Hip fractures are a common cause of mortality in elderly patients. This study aimed to determine the predictive factors affecting mortality among patients over the age of 80 who underwent surgical treatment for hip fractures.

**Methods:**

We searched the Turkish Ministry of Health’s e-health database to identify patients over 80 years old who had undergone surgery for proximal femoral fractures from 2016 to 2022. This process yielded 53,495 patients. Demographic data as well as comorbidities, blood transfusions, postoperative 90 days medical complications, and mortality was investigated. Multivariate logistic regression analysis was performed to identify risk factors for one year mortality in patients undergoing surgical treatment for proximal hip fractures.

**Results:**

The mortality rate was 37.2% in the first year. The mean Charlson comorbidity index(CCI) was 6.8 (range: 4–22). In the postoperative period, 68.6% of the patients received blood transfusions. Logistic regression analysis identified significant predictors of one-year mortality in surgical patients, including male gender, increased age, higher CCI scores, AKI, PE, pneumonia, electrolyte imbalance, gastrointestinal bleeding, blood transfusion, and increased mortality risks with hemiarthroplasty and internal fixation compared to total hip arthroplasty. (*p* < 0.001 for all).

**Conclusions:**

This large cohort study demonstrated that the mortality rate is high and that the type of surgery, male gender, older age, blood transfusion requirements, and high CCI score are associated with mortality in patients over 80 years of age who have undergone surgery for hip fractures. Preoperative optimization and postoperative care are critical for these vulnerable elderly patients.

## Introduction

Hip fractures are an important cause of morbidity and mortality in the geriatric age group. Geriatric hip fractures usually develop after minor traumas due to the facilitating effect of osteoporosis [1]. Advancing technology and new treatment modalities have increased life expectancy and the population over the age of 65 has grown, particularly in developed and developing countries, and it is expected to grow much more in the future. According to the World Health Organization, approximately 500 million people were 65 years old or older in 2010, accounting for 8% of the global population. This figure is anticipated to increase to roughly 1.5 billion by 2050, accounting for 16% of the world’s population [2].

The incidence of hip fractures worldwide is predicted to increase from 1.66 million in the early 1990s to approximately 6.26 million by 2050 [3]. Hip fractures have a high mortality rate among older adults, especially those aged > 80 years [4]. The presence of hip fractures in these patients increases mortality by approximately three times compared to the normal population [5]. Treatment options for hip fractures are internal fixation (IF), hemiarthroplasty (HA), or total hip arthroplasty (THA) [6, 7]. The incidence of postoperative complications is relatively high in patients undergoing surgery for hip fractures, and the mortality rate within one year after surgery ranges from 14 to 36% [8].

Previous studies have identified predictive factors affecting mortality after hip fractures, such as age, type of surgery, time to surgery, blood transfusion requirements, gender, underlying diseases, use of anticoagulants, and hospitalization time [9, 10]. Although many factors that increase the risk of mortality have been identified, there is no consensus in the literature on these factors. A better understanding of risk factors for mortality may allow for comprehensive optimization before surgery and meticulous perioperative care to reduce mortality among these highly vulnerable elderly patients.

This study aimed to analyse the e-health database of the Turkish Ministry of Health and determine the predictive factors for mortality among patients over the age of 80 who underwent surgical treatment for proximal hip fractures.

## Materials and methods

### Study setting

The health records of every citizen in Türkiye are gathered via a digital health platform, namely the e-Nabız e-health database, which is affiliated with the Turkish Ministry of Health. This comprehensive database contains data collected from all public, private, and university healthcare institutions across Türkiye [11]. The study was conducted according to the Declaration of Helsinki and received approval from the Turkish Ministry of Health, with a waiver of informed consent for retrospective data analysis and the health information privacy law (ID: 95741342-020/27112019). The research protocol also received approval from the Turkish Ministry of Health, which granted a waiver of informed consent for the retrospective analysis of data. No direct contact with participants and data were collected anonymously and retrospectively.

## Data collection and study population

To create the study population, patients with proximal femur fractures were initially identified using the International Classification of Diseases Tenth Revision (ICD-10) codes of S72.0 (femoral neck fracture), S72.1 (intertrochanteric femur fracture). Patients aged ≥ 80 years who underwent IF, HA, or THA from 2016 to 2022 were then identified using operation-specific procedure codes (https://skrs.saglik.gov.tr/) Patients under 80 years of age, those who received conservative treatment and patients with subtrochanteric femur fracture were not included to the study. All of the outcome variables were extracted and collected from patient medical records stored in the e-Nabız database of the Turkish Ministry of Health.

Outcome Measures.

### Descriptive variables

Patient-related variables included age at the time of the operation, gender, body mass index (BMI; kg/m^2^), and preoperative comorbidities. Comorbidities were classified according to the Charlson comorbidity index (CCI) described by Charlson et al. [12]. Surgery-related variables were also documented, including the type of operation (IF, HA, or THA). Postoperative 90 days medical complications were classified according to ICD-10 codes.

### Mortality-related variables

Postoperative mortality rates after primary surgery at one week, at three months, at one year, and overall from 2016 to 2022 were estimated for the entire study population. Overall mortality was defined as cases of mortality occurring from the day of the patient’s surgery until the survey was conducted on 01 January 2024.

### Statistical analysis

Data were analysed using IBM SPSS Statistics 25.0 (IBM Corp., Armonk, NY, USA). The normality of the data was evaluated using skewness and kurtosis values. These statistical measures provided insight into the symmetry of the data distribution, allowing for the determination of whether data conformed to normal distribution. Quantitative variables were expressed as mean ± standard deviation. Qualitative variables were expressed as numbers (n), frequencies, or ratios. Categorical variables were compared using the Pearson chi-square test. Parametric variables were compared using the independent sample t-test.

A logistic regression model was employed to ascertain the variables influencing the likelihood of mortality within a one year timeframe. To identify factors influencing mortality over the course of one year, we initially performed univariate analysis using logistic regression. Subsequently, multivariate logistic regression analysis was conducted, incorporating the factors that exhibited statistical significance in the univariate analysis.

Values of *p* < 0.05 were considered to indicate statistical significance. The Bonferroni correction is applied when multiple comparisons are conducted to reduce the risk of Type I errors (false positives).

## Results

We retrospectively analysed 53,495 patients (67% female) over 80 years of age who were operated on for hip fractures between 2016 and 2022. The mean age was 86 (range: 80–114) years, and 77.6% (*n* = 41,514) of the patients had a femoral neck fracture, 22.4% (*n* = 11,981) had a pertrochanteric fracture. HA was performed for 66.3% (*n* = 35,462) of the patients, IF was performed for 30.5% (*n* = 16,319), and THA was performed for 3.2% (*n* = 1,714). (Table 1)

Postoperative complications in 90 days after surgery are summarized in Table 2. The mean LOS was 7.4 (range: 0–21) days. The mean age-adjusted CCI score was 6.8 (range: 4–22). The rate of perioperative blood transfusion was found to be 68.6% (*n* = 36,699 patients).The mortality rate was 2.8% (*n* = 1,505) in the first week, 21.8% (*n* = 11,679) in the first three month, and 37.2.% (*n* = 19,912) in the first year. Furthermore, we observed an increase in mortality as age advanced (Table 3). The mean time to postoperative mortality was 513.6 (range: 0–2903) days. Among patients aged 80–90 years, the mean time to death was 546 days (range: 0–2.864 days), while for those over 90 years old, the mean time was 417 days (range: 0–2,903days), with a statistically significant difference (*P* < 0.001). One-year mortality rates are significantly higher in males (*n* = 7889, 44.7%) compared to females (*n* = 12023, 33.5%) with a P value < 0.001.

**Table 1:** Types of fractures and Surgical Procedure

Surgical Procedure	Femoral neck fracture		Intertrochanteric fracture	Total
Internal Fixation *(% within etiology)*	9531 (23)		6788 (56.7)	16,319(30.5%)
Hemiarthroplasty *(% within etiology)*	30,517 (73.5)		4945 (41.3)	35,462 (66.3%)
THA *(% within etiology)*	1466 (3.5)		248 (2.1)	1714 (3.2%)
Total	41,514(100%)		11,981 (100%)	53,495 (100%)

HA: Hemiarthroplasty; THA: Total Hip Arthroplasty

**Table 2:** Mortality rates and 90 days medical complications regarding the type of surgery

	Internal Fixation	Hemiarthroplasty	Total Hip Arthroplasty	*P* Value
	*n* (%)	*n* (%)	*n* (%)	
Blood Transfusion*	10,590 (64.9)	24,938 (70.3%)	1171 (68.3)	**0.001**
AKI	1015 (6.2)	2374 (6.7%)	97 (5.7)	0.043
Arithmia	558 (3.4)	1297 (3.7)	54 (3.2)	0.25
PE	564 (3.5)	1157 (3.3)	57 (3.3)	0.52
MI	167 (1)	403 (1.1)	30 (1.8)	0.22
Pneumonia	1044 (6.4)	2466 (7)	145 (8.5)	**0.002**
Electrolyte Imbalance	586 (3.6)	1375 (3.9)	74 (4.3)	0.15
GI Bleeding	275 (1.7)	2692 (2)	26 (1.5)	0.07
UTI	888 (5.4)	1997 (5.6)	92 (5.4)	0.68
Mortality in one week	369 (2.3)	1101 (3.1)	1679 (3.5)	**< 0.001**
Mortality in three months	3398 (20.8)	7964 (22.5)	317 (18.5)	**< 0.001**
Mortality in one year	5911 (36,2)	13,457 (37.9)	544 (31.7)	**< 0.001**

AKI: Acute kidney injury, VTE: venous thromboembolism, PE: pulmonary embolism, MI: myocardial infarction; GI: gastrointestinal, UTI: urinary tract infection

*Intraoperative and in post-operative 10 days

Bonferroni correction applied. Significance is determined in bold, if *P* < 0,016

**Table 3:** Mortality rates according to Age groups

	Age		*P* value
	80–89	90**≤**	
Mortality in 10 days, n (%)	1017 (2.4%)	488 (4.1%)	**< 0.0001**
Mortality at 3 month, n (%)	8105 (19.3)	3574 (30.5)	**< 0.0001**
Mortality at 1 year, n (%)	14,214 (34)	5698 (48.7%)	**< 0.0001**
Overall mortality, n (%)	27,200 (65.1)	9170 (78.5)	**< 0.0001**

Significance is determined in bold if *P* < 0.05

**Table 4:** Univariate and Multivariate Logistic regression analyses for defining predictors of one-year mortality in patients undergoing surgery for hip fractures

	Univariate logistic regression		Multivariate logistic regression	
	Odds Ratio(OR)(95% CI)	*P* value	Adjusted Odds ratio (OR) (95% CI)	*P* value
Sex(Reference: Female)	1.6 (1.54–1.66)	**0.001**	1,66 (1.6-1,73)	**0.001**
Age	1.85 (1.77–1.92)	**0.001**	1.93 (1.85–2.01)	**0.001**
CCI	1.37 (1.31–1.42)	**0.001**	1.38 (1.3–1.43)	**0.001**
AKI	4 (3.8–4.4)	**0.001**	3.1 (2.87–3.36)	**0.001**
PE	2.63 (2.4–2.9)	**0.001**	2.05 (1.85–2.27)	**0.001**
Pneumonia	3 (2.8–3.25)	**0.001**	2.33 (2.16–2.51)	**0.001**
Electrolyte imbalance	3.51 (3.2–4.2)	**0.001**	2.42 (2.2–2.68)	**0.001**
GI bleeding	3.67 (3.2–4.2)	**0.001**	2.93 (2.55–3.38)	**0.001**
UTI	1.26 (1.17–1.35)	**0.001**	0.97 (0.89–1.05)	0,5
Blood Transfusion	1.25 (1.2–1.3)	**0.001**	1.21 (1.16–1.26)	**0.001**
Surgery Type (reference: THA)			1	
HA	1.22 (1.1–36)	**0.001**	1.22 (1.01–1.37)	**0.001**
IF	1.31 (1.19–1.46)	**0.001**	1.3 (1.67–1.45)	**0.001**

Bonferroni correction is applied. Significance is determined in bold, if *P* < 0.004

The logistic regression analyses identified several significant predictors of one-year mortality in patients undergoing surgery. Males had a higher mortality risk compared to females (OR: 1.66, 95% CI: 1.60–1.73, *P* = 0.001). Increased age (OR: 1.93, 95% CI: 1.85–2.01, *P* = 0.001) and higher CCI scores (OR: 1.38, 95% CI: 1.30–1.43, *P* = 0.001) were also associated with higher mortality. Complications such as AKI (OR: 3.10, 95% CI: 2.87–3.36, *P* = 0.001), PE (A OR: 2.05, 95% CI: 1.85–2.27, *P* = 0.001), pneumonia (OR: 2.33, 95% CI: 2.16–2.51, *P* = 0.001), electrolyte imbalance (OR: 2.42, 95% CI: 2.20–2.68, *P* = 0.001), and gastrointestinal bleeding (OR: 2.93, 95% CI: 2.55–3.38, *P* = 0.001) were significant predictors. Blood transfusion (OR: 1.21, 95% CI: 1.16–1.26, *P* = 0.001) showed a slight increase in mortality risk, while UTI did not significantly affect mortality (OR: 0.97, 95% CI: 0.89–1.05, *P* = 0.5). Compared to THA, hemiarthroplasty and internal fixation were associated with increased mortality risk (OR for HA: 1.22, 95% CI: 1.01–1.37, *P* = 0.001; OR for IF: 1.30, 95% CI: 1.16–1.45, *P* = 0.001). (Table 4)

When the relationship between type of surgery and mortality was investigated, it was found that mortality was statistically significantly highest among patients undergoing hemiarthroplasty, followed by IF, and lowest among patients with total hip replacements (*P* = 0.001). (Table 2) However, type of fracture didn’t have a significant effect on one year mortality in univariate logistic regression (*p* = 0,512). Therefore it’s not included in the multivariate logistic regression.

## Discussion

This large national database study demonstrated that male gender, blood transfusion requirements, older age, and high CCI score and having complications within 90 days following surgery are predictors for mortality in patients who are over 80 years old and undergoing surgical treatment for proximal hip fractures. Mortality rates in this patient population tend to be higher when IF or HA is applied compared to THA.

Hip fracture is a major cause of morbidity and mortality in the elderly population. Several studies reported high mortality rates (8.4–58%) at one year of follow-up among patients over 80 years old [9, 13, 14]. Mortality rates increased by nearly three times when compared to the patient group below the age of 80 [5, 15]. In the present study, we found a one year mortality rate of 37.2% in the Turkish patient population above 80 years. However, among above 90 years olders, the mortality rate was approximately 1.5 times higher compared to octogenarians (48.7% and 34%, respectively) (Table 3). The increase in age-related comorbid factors and decreased body resistance may account for this difference.

The choice of surgery after hip fracture for patients over 80 years of age is a crucial decision that can impact patient outcomes, including mortality rates. HA and THA provide the advantage of early mobility, but they require larger exposure. On the other hand, intramedullary nailing may be performed with minimally invasive techniques, which leads to several advantages such as less blood loss and shorter operation time [16, 17]. However, the time to full weight-bearing may be prolonged. High failure rates are another concern for IF [18]. The relationship between different treatment options and mortality has been studied before, and several systematic reviews emphasize that there is no association between type of surgery and one year mortality [19, 20]. A meta-analysis examining randomized controlled trials concluded that IF techniques such as dynamic hip screws and proximal femoral nailing had a higher risk of failure compared to HA. However, the mortality rate was found to be higher among HA patients [21]. On the other hand, there are many studies that suggest lower mortality rates among patients undergoing IF compared to those undergoing arthroplasty [7, 9, 22, 23]. In our cohort, the lowest mortality rate at the first year was observed for THA, which was followed by IF and HA, respectively, at 31.7%, 36.2%, and 37.9%. It is important to remember that the relationship between surgical technique and mortality in hip fractures is multifactorial. The higher mortality rate in the HA group may be explained by older patients receiving HA and the greater need for blood transfusions in that group of patients. The low mortality rate with THA may be attributed to the utilization of THA for relatively healthy and active patients.

Gender is another factor that has been studied as a prognostic indicator for hip fractures in elderly patients. Male gender was previously found to be associated with a higher mortality rate in elderly patients with hip fractures at respective rates of 27.8% versus 20.1% for patients between 80 and 84 years and 34.7% versus 25% for patients older than 85 years [24]. However, there have been other studies with conflicting results. A Spanish cohort of 26,000 hip fracture patients older than 75 years found that female gender was associated with mortality in hip fractures [25]. On the other hand, there are also reports stating that gender has no effect on mortality [9]. In our study, although the majority of hip fracture patients were female, we observed a higher mortality rate among male patients (44.7% vs. 33.5%). Higher CCI scores among men may explain the higher mortality rates. Further studies are needed to clarify the role of gender as a prognostic factor for hip fractures in elderly patients. It is known that men generally have lower rates of osteoporosis and better bone quality than women in the same age group [26]. Based on this, we believe that when hip fractures occur in men, they may be associated with an underlying, more serious medical condition or a higher degree of trauma, which could potentially influence mortality.

Among patients undergoing hip surgery following a fracture, blood transfusion rates have been reported to range between 28% and 75% [7, 27, 28]. However, postoperative blood transfusions following geriatric hip fractures are closely associated with postoperative complications and mortality. Additionally, higher rates of mortality were reported in patients who needed two or more blood transfusions [9]. Furthermore, transfusion rates differ among surgical procedures. IF is known as an advantageous surgical method in the treatment of hip fractures due to its short surgical time and minimal blood loss [1]. Parker et al. revealed higher needs for transfusion among HA patients compared to IF patients [7]. In our study, 68.6% of the patients received blood transfusions. Additionally, consistent with the literature, the fewest blood transfusions were administered to the patients who underwent IF (64.9%). Many factors, such as preoperative anaemia, gender, fracture type, type of surgery, and operation time, can affect the need for blood transfusions. Our blood transfusion rate is high compared to previous studies and there are several possible reasons for this. Adequate preoperative optimization is not always possible for patients with hip fractures due to accompanying anaemia, chronic renal failure, or hepatic diseases. Preoperative anaemia is the main reason for reaching the blood transfusion threshold. Furthermore, most patients (70%) in our cohort underwent HA or THA, which may increase the requirements for blood transfusion. Elderly patients have many comorbidities that may increase the risk of bleeding. Using potent anticoagulants before the fracture and after the surgery for venous thromboembolism prophylaxis may increase the bleeding. Finally, the low threshold for blood transfusion in elderly patients may be another reason for the high transfusion rate.

The CCI is a widely used tool for assessing the burden of patients’ comorbidities. It quantifies the presence and severity of various comorbid conditions and provides an overall score that predicts the risk of mortality and other adverse outcomes. A higher preoperative CCI score is associated with higher postoperative mortality [12]. Although there is no consensus on a cut-off value for the CCI, there are studies reporting that CCI values of ≤ 3 are associated with a better prognosis [1, 5, 29]. In our study, similar to previous studies in the literature, we observed high mortality rates among patients with high CCI scores.

Postoperative complications are among the significant factors that increase mortality following hip fractures. Studies have shown that the development of urinary system issues, respiratory and cardiovascular system problems, and electrolyte imbalances in the postoperative period contributes to increased mortality [8, 14, 19, 29, 30]. Many of these complications, especially those that elevate mortality and length of hospital stay, are critical risk factors [19, 31]. Similarly, our study found that the occurrence of acute kidney injury, pulmonary embolism, pneumonia, electrolyte imbalances, and gastrointestinal bleeding increased mortality by two to three times. These results underscore the importance of preventing complications and emphasizing perioperative patient care to reduce mortality.

The main strengths of the present study are the large number of patients included and the ability to obtain data on all deaths, both in-hospital and out-of-hospital, such as those occurring at home, for a large cohort of nearly 55,000 patients. This study contains one of the largest cohorts to date, revealing the predictive factors for mortality after hip fractures in patients over 80 years of age. However, there are also some limitations. The main limitation is information bias since the research was based on data retrospectively obtained from a national database. The influence of confounding variables and selection bias cannot be ruled out in studies with larger patient populations. Furthermore, this study did not include laboratory values or physiological variables, such as the use of anticoagulants. ICD-10 codes were used to obtain information on complications, and no detailed records on disease severity were reviewed; therefore, misclassification and missing data may be an issue. Additionally, there was no clear indication for treatment choice (THA, IF, HA), so variations in the surgeon’s knowledge and experience could have influenced the outcomes. Mortality was not stratified according to the surgeons’ volume and experience. We could not identify specific causes of death, and functional results were not evaluated. These are important considerations, as knowing the cause of death can help explain the mechanism of mortality. Due to the legal regulations in our country, although mortality data is highly reliable (yes/no/time of death), we are unable to obtain information on whether the deaths occurred at home, during a later admission to another hospital, or in a nursing home.

This study demonstrated that the one year mortality rate of patients aged over 80 and undergoing surgery after hip fractures is high (34.9%). The type of surgery, male gender, older age, blood transfusion requirements, and high CCI scores were associated with mortality. The elevated risk of mortality after hip fracture in the elderly population should be recognized. Patients should be optimized regarding all modifiable risk factors. Patients and their families should be counselled accordingly. Team management including the participation of a geriatrician may be beneficial. Future research should entail prospective studies with comprehensive evaluation, optimization, and management of this patient group to reduce the risk of mortality and further elucidate the complex relationships between these factors and mortality in cases of hip fractures.

## Data Availability

The data that support the findings of this study are available from the corresponding author upon reasonable request. However, the dataset is derived from the e-Nabız (National Electronic Health Record) system and cannot be shared publicly due to privacy and confidentiality restrictions.

## Declarations

### Generative AI and AI-assisted technologies in the writing process

During the preparation of this work the authors used ChatGPT in order to edit language. After using this tool/service, the authors reviewed and edited the content as needed and take full responsibility for the content of the publication.
